# Designing equity-focused sanitation policies in Sub-Saharan Africa: the critical role of subnational data

**DOI:** 10.1186/s12889-025-25862-8

**Published:** 2025-12-02

**Authors:** Alpha Umaru Bai-Sesay, Baba Bangura, Gassimu Bai-Sesay, Jusu Musa

**Affiliations:** 1 Ministry of Health, National Public Health Agency, Freetown, Sierra Leone; 2 Research and Scientific Division, Sustainable Health Systems, Freetown, Sierra Leone; 3 Insights for Development Impact, Freetown, Sierra Leone; 4 The Conservation Society of Sierra Leone, Freetown, Sierra Leone

**Keywords:** Equity metrics, Open defecation, Rural-urban disparities, Sanitation inequities, SDG 6.2, Sub-Saharan africa, sanitation policies

## Abstract

**Background:**

Open defecation remains a major public health and equity challenge in Sub-Saharan Africa, where national averages often mask profound subnational disparities. Progress toward Sustainable Development Goal (SDG) 6.2 depends not only on increasing coverage but on closing persistent rural-urban gaps. This study quantified the magnitude and national consequences of these inequities using harmonized 2022 data.

**Methods:**

We analyzed WHO/UNICEF Joint Monitoring Programme (JMP) 2022 estimates for 20 low- and lower-middle-income countries using four standard inequality metrics: the absolute difference (D), rate ratio (R), population attributable risk (PAR), and population attributable fraction (PAF). These indicators quantify both the scale of rural-urban gaps and the portion of the national burden attributable to rural disadvantage. Countries were grouped into a three-tier equity typology based on national prevalence and the size of the rural-urban gap.

**Results:**

National open-defecation prevalence ranged from 0.1% (Gambia) to 67.0% (Eritrea), with rural rates consistently and substantially higher than urban rates. In most countries, rural residents were three to ten times more likely to practise open defecation than urban residents. PAR and PAF results showed that eliminating rural disadvantage would reduce national prevalence by up to 40–60% points in the most unequal settings. Countries clustered into three profiles: high burden with extreme inequity (*n* = 6), moderate burden with persistent inequity (*n* = 10), and low burden with residual inequity (*n* = 4). We found that eliminating the rural disadvantage could reduce the national prevalence of open defecation by up to 40% points in the most unequal settings, demonstrating that equity is central to achieving SDG 6.2.

**Conclusions:**

Open defecation in Sub-Saharan Africa is overwhelmingly concentrated in rural populations, making inequity, not simply low coverage, the central barrier to achieving SDG 6.2. Integrating simple equity metrics such as D, R, PAR, and PAF into national monitoring systems can help governments identify priority populations, estimate potential gains from reducing disparities, and target investments more efficiently and fairly. Equity-focused sanitation strategies are therefore essential to ensuring that no community is left behind.

## Introduction

Open defecation remains both a public-health hazard and a matter of human dignity and safety. Beyond its association with faecal-oral diseases, lack of sanitation undermines privacy and disproportionately exposes women and children to harassment and infection [1, 2]. Despite global commitments under Sustainable Development Goal (SDG) 6.2 to end open defecation by 2030, progress in Sub-Saharan Africa has been uneven and inequitable [3]. According to the 2023 WHO/UNICEF Joint Monitoring Programme (JMP), more than 180 million people in the region still practice open defecation over one-third of the global total [4].

Rural-urban disparities stand out as one of the most pervasive and consequential dimensions of inequity. In Chad, Niger, and South Sudan, more than 70% of rural populations practiced open defecation in 2022, compared with fewer than 20% of their urban counterparts [5, 6]. Such gaps reveal not only a disparity in coverage but also a structural exclusion of rural populations from the benefits of development [7]. National averages, often used in monitoring and policy evaluation, obscure these inequities and risk reinforcing them [8]. For example, Chad’s national prevalence of open defecation (62.6%) masks the far higher burden in rural communities (77.6%), a distinction critical for targeted policy action. Without disaggregated analyses, resource allocation may remain skewed toward urban centers, perpetuating cycles of rural disadvantage [9]. These inequities persist not only because of poverty and remoteness but also due to weak decentralization, limited community engagement, and socio-cultural norms that shape latrine adoption. Inequity, in this context, refers to systematic and avoidable differences in sanitation access between population groups that are considered unfair and modifiable through policy action.

Despite recognition of these inequities, most cross-country analyses of sanitation in Sub-Saharan Africa remain descriptive, relying on national or occasionally subnational prevalence figures without systematically applying equity-sensitive measures [10] Although national monitoring systems routinely report average coverage, such aggregates obscure subnational disparities that drive health and social inequities. The WHO/UNICEF Joint Monitoring Programme (JMP) provides disaggregated data, but few studies translate these data into standardized inequality metrics that can quantify both the magnitude and public health consequences of disparities [8]. Metrics such as absolute difference (D), ratio (R), population attributable risk (PAR), and population attributable fraction (PAF) are widely endorsed in health equity research yet remain underutilized in the sanitation sector. This omission has limited the ability of governments and development partners to identify priority populations, estimate the proportion of national burden attributable to inequity, and design interventions that are both efficient and just.

Despite recognition of these disparities, research linking inequality measurement to policy differentiation remains limited. Addressing this analytical gap can guide countries toward equity-sensitive strategies for achieving universal sanitation. Achieving SDG 6.2 is not simply a matter of aggregate progress but of equitable progress. Rural populations, who shoulder the overwhelming share of open defecation burden, require tailored interventions that extend beyond infrastructure provision to include community mobilization, culturally grounded behavior change, and long-term systems strengthening [11]. Equity metrics offer more than diagnostic clarity; they provide a roadmap for action, quantifying the potential gains from eliminating disparities and enabling policymakers to prioritize investments with maximal impact [12].

This study aimed to quantify the magnitude and burden of rural-urban inequities in open defecation across 20 Sub-Saharan African countries using harmonized 2022 JMP data and standardized equity indicators to inform evidence-based, equity-focused policy design.

## Methods

### Study design and setting

We conducted a retrospective, cross-sectional analysis of subnational inequities in open defecation across 20 low- and lower-middle-income countries in Sub-Saharan Africa in 2022. The study focused on disparities between rural and urban populations, reflecting the region’s entrenched geographic divide in sanitation infrastructure. Countries included were Sierra Leone, Burkina Faso, Burundi, the Central African Republic, Chad, the Democratic Republic of Congo, Eritrea, Ethiopia, Gambia, Guinea-Bissau, Liberia, Madagascar, Malawi, Mali, Mozambique, Niger, Rwanda, South Sudan, Togo, and Uganda. These countries represent diverse geographic and socioeconomic contexts with persistent sanitation challenges.

Countries were included if they had 2022 WHO/UNICEF JMP estimates disaggregated by rural and urban residence available in the WHO Health Inequality Data Repository (HEAT, version 6.0). Countries lacking disaggregated or complete 2022 data were excluded. The final sample represents approximately 516 million people across Sub-Saharan Africa, capturing a range of geographic, economic, and policy contexts and providing a regionally representative picture of sanitation inequities.

### Data sources and variables

The primary outcome was the prevalence of open defecation, defined by the WHO/UNICEF Joint Monitoring Programme as the disposal of human feces in open without the use of a sanitation facility. Data was obtained from the WHO/UNICEF JMP database, accessed via the WHO Health Inequality Data Repository (HEAT, version 6.0, June 2025). The JMP compiles nationally representative estimates derived from standardized household surveys and population censuses. Estimates are harmonized using consistent definitions and interpolation protocols. The equity stratifier was place of residence (urban vs. rural), defined according to national census classifications and standardized within JMP. The country-level prevalence data used for analysis are summarized in Table 1.

**Table 1:** Prevalence of open defecation by place of residence, selected countries in Sub-Saharan Africa (2022)

Country	Rural Estimate (%)	Urban Estimate (%)	Rural Population	Urban Population
Sierra Leone	26.0	4.2	4833746.0	3771972.0
Burkina Faso	46.7	5.6	15446047.0	7227715.0
Burundi	1.7	0.1	11031286.0	1858290.0
Central African Republic	38.9	6.8	3173417.0	2405727.0
Chad	77.6	15.4	13458554.0	4264761.0
Democratic Republic of the Congo	18.9	3.9	52636800.0	46373412.0
Eritrea	88.7	32.9	2058647.0	1306640.0
Ethiopia	21.8	3.3	95420800.0	27959126.0
Gambia	0.0	0.1	978162.0	1727830.0
Guinea-Bissau	15.0	0.4	1157198.0	948368.0
Liberia	57.3	15.7	2488814.0	2813868.0
Madagascar	44.9	16.7	17801970.0	11809744.0
Malawi	2.9	1.1	16736441.0	3668876.0
Mali	7.4	1.2	12327740.0	10265850.0
Mozambique	28.5	5.2	20379448.0	12590070.0
Niger	76.3	9.6	21780402.0	4427576.0
Rwanda	2.0	1.3	11335329.0	2441369.0
South Sudan	73.3	8.1	8638206.0	2274958.0
Togo	61.5	11.4	4962262.0	3886437.0
Uganda	4.6	1.8	34889564.0	12360019.0

*Source: WHO/UNICEF Joint Monitoring Programme (JMP)*,* accessed via WHO Health Inequality Data Repository (HEAT v6.0)*

National estimates were derived from standardized household surveys and population censuses harmonized by JMP. All data were cross verified against country profiles in the JMP database to confirm accuracy prior to analysis.

### Measures of inequality

We analyzed four equity metrics generated by HEAT that are recommended for health inequality monitoring: Absolute difference: the absolute percentage point gap in open defecation prevalence between rural and urban populations (D = P_rural - P_urban). Rate ratio (R): the ratio of rural-to-urban prevalence (R = P_rural/P_urban). Population Attributable Risk (PAR): the absolute reduction in national prevalence that would occur if all subgroups had the same prevalence as the subgroup with the lowest prevalence. Population Attributable Fraction: the proportion of the national burden of open defecation attributable to rural-urban inequity, calculated as (PAR ÷ national prevalence) × 100.

By convention, the WHO HEAT tool defines the reference group as the population with the lowest prevalence. Under this specification, PAR and PAF values for open defecation are often returned as negative numbers, indicating “excess” burden in disadvantaged groups. For interpretability and comparability, we reported positive magnitudes of PAR and PAF, reflecting the scale of inequity rather than its direction. This approach is widely used in equity research to communicate the size of disparities in an intuitive manner.

### Development of equity typology

To translate the inequality metrics into a policy-relevant classification, we developed a transparent, rule-based equity typology that incorporates two dimensions: (1) the national burden of open defecation and (2) the magnitude of rural–urban inequity. The typology was constructed through a three-step operational process designed to enhance clarity, reproducibility, and policy relevance.

- Step 1: Selection of core dimensions: Two indicators were used to differentiate countries: (a) National burden: the overall national prevalence of open defecation. (b) Inequity magnitude: the absolute difference (D) between rural and urban prevalence estimates.
- Step 2: A priori definition of thresholds: Thresholds were established before the analysis, drawing on benchmarks commonly used in WHO and African Sanitation Policy Guideline frameworks. Countries were classified into one of three categories: (a) High burden/extreme inequity: (i) National prevalence ≥ 30%, and (ii) Rural–urban absolute difference ≥ 40% points. (b) Moderate burden/persistent inequity: (i) National prevalence 10–29.9%, and (ii) Rural–urban difference ≥ 15% points. (c) Low burden/residual inequity: (i) National prevalence < 10%, and (ii) Rural–urban difference < 15% points.

These thresholds reflect policy-anchored criteria, not empirical cut-points, and are intended to guide differentiated national strategies.

- Step 3: Assignment of countries: Each country was placed into the category for which it met both criteria. This deterministic approach ensured that classification was fully transparent and reproducible, without subjective weighting or post-hoc adjustments. Final groupings are presented in Table 3.

This structured typology enhances comparability across countries and supports the use of equity metrics as practical decision-support tools in national sanitation planning.

### Data handling and analysis

We extracted national, urban, and rural prevalence values along with HEAT-generated estimates for D, R, PAR, and PAF. All outputs were imported into Microsoft Excel (version 16.76, Microsoft Corporation, Redmond, WA, USA) for cleaning, tabulation, and visualization.

To aid interpretation, we classified countries into a rule-based typology reflecting both national open-defecation burden and magnitude of rural–urban disparity. Thresholds were defined a priori as follows: (1) High burden/extreme inequity: national prevalence ≥ 30% and absolute difference ≥ 40% points; (2) Moderate burden/persistent inequity: national prevalence 10–29.9% and difference ≥ 15% points; (3) Low burden/residual inequity: national prevalence < 10% and difference < 15% points. These thresholds align with policy-relevant sanitation benchmarks used by WHO and the African Sanitation Policy Guidelines, enabling comparability across contexts.

Uncertainty intervals originally generated by the WHO HEAT tool were reviewed for completeness and validity. However, in several countries the exported files contained identical lower and upper bounds (e.g., “−75.5 to − 75.5”), indicating placeholder rather than genuine interval estimates. Because these values were not statistically meaningful, all uncertainty intervals were excluded to prevent misinterpretation. As this study is descriptive and not inferential, point estimates were retained as the most transparent representation of the data.

#### Ethics

This study used publicly available, de-identified secondary data from the WHO/UNICEF JMP database accessed through HEAT. No individual-level data were analyzed, and institutional ethics approval was not required. Clinical trial number: not applicable.

## Results

**Fig. 1:**
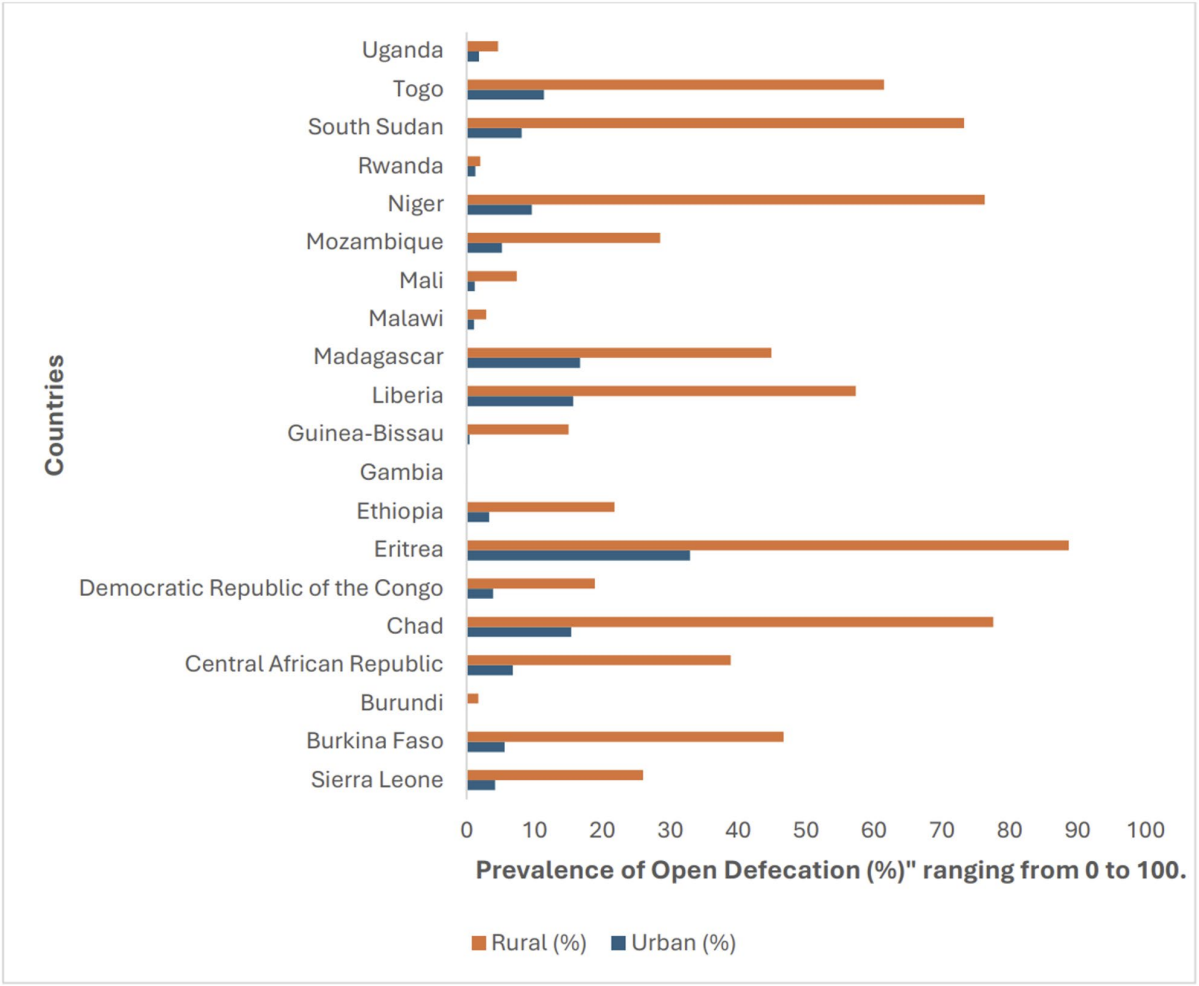
Country-specific rural-urban disparities in open defecation prevalence across 20 Sub-Saharan African countries, 2022

Nationally, open defecation remained highly prevalent across much of Sub-Saharan Africa in 2022, with wide disparities between rural and urban populations Fig. 1.

Across the 20 countries analyzed, national prevalence ranged from 0.1% in The Gambia to 67.0% in Eritrea, while the median rural-urban difference exceeded 40% points. Most countries exhibited rural prevalence rates more than three times higher than urban levels, confirming that rural disadvantage continues to drive the regional sanitation burden (Table 1).

Absolute differences (D) ranged widely, from less than 5% points in The Gambia, Rwanda, and Malawi to more than 70 points in Eritrea, Chad, and South Sudan (Table 2). Rate ratios (R) followed a similar gradient, indicating that in the most unequal settings rural residents were over 10 times more likely to practise open defecation than their urban counterparts. Population-attributable risks (PAR) and population-attributable fractions (PAF) underscored the scale of inequity: in several countries more than half of the national burden of open defecation was attributable to rural disadvantage. These indicators collectively show that closing rural–urban gaps would yield large national gains in sanitation coverage, directly advancing SDG 6.2.

**Table 2:** Rural–urban inequalities in open defecation across 20 Sub-Saharan African countries, 2022

Country	National prevalence (%)	D (pp)	*R* (rural/urban)	PAR (pp)	PAF (%)
Sierra Leone	16.4	21.7	6.2	12.2	74.3
Burkina Faso	33.6	41.1	8.4	28	83.5
Burundi	1.4	1.6	21.1	1.4	94.5
Central African Republic	25	32	5.7	18.2	72.7
Chad	62.6	62.2	5.1	47.2	75.5
Democratic Republic of Congo	11.8	14.9	4.8	7.9	67.1
Eritrea	67	55.8	2.7	34.1	50.9
Ethiopia	17.6	18.6	6.7	14.4	81.4
Gambia	0.1	0.1	0	0	0
Guinea-Bissau	8.4	14.6	36	8	95.1
Liberia	35.2	41.6	3.6	19.5	55.4
Madagascar	33.6	28.1	2.7	16.9	50.3
Malawi	2.6	1.8	2.7	1.5	57.7
Mali	4.5	6.2	6.4	3.4	74.8
Mozambique	19.6	23.3	5.5	14.4	73.4
Niger	65	66.7	8	55.4	85.2
Rwanda	1.9	0.7	1.6	0.6	32.3
South Sudan	59.7	65.2	9.1	51.6	86.5
Togo	39.5	50.1	5.4	28.1	71.2
Uganda	3.8	2.8	2.6	2.1	53.8

*WHO/UNICEF Joint Monitoring Programme (JMP)*,* accessed via WHO Health Inequality Data Repository (HEAT v6.0).*

A few outliers merit clarification. In The Gambia, both rural and urban prevalence were near zero (0.0% rural, 0.1% urban), producing a negligible absolute difference despite near-elimination of open defecation nationally. In Rwanda, the relatively low PAF (≈ 32%) reflects the country’s already low national prevalence and minimal absolute gap rather than methodological error. These exceptions confirm that the inequality metrics behave consistently with the underlying epidemiology.

Using predefined thresholds, three equity profiles emerged (Table 3). High-burden/extreme-inequity countries: *Eritrea*,* Chad*,* South Sudan*,* Niger*,* Burkina Faso*,* Central African Republic*, exhibit high national prevalence (≥ 30%) and deep rural disadvantage (D ≥ 40% points). In these contexts, rural open defecation remains a population-wide norm requiring large-scale rural infrastructure investment, governance reform, and national sanitation campaigns. Moderate-burden/persistent-inequity countries: *Madagascar*,* Malawi*,* Mali*,* Mozambique*,* Liberia*,* Sierra Leone*,* Ethiopia*,* Togo*,* Guinea-Bissau*,* and DRC*, show national prevalence between 10% and 30% with sustained rural–urban gaps above 15 points. These settings need targeted subnational programmes combining behaviour-change communication and equity-focused resource allocation. Low-burden/residual-inequity countries: *Rwanda*,* Burundi*,* Gambia*,* and Uganda*, have achieved near-universal progress but retain small inequities that must be addressed to ensure that no groups are left behind.

**Table 3:** Typology of Rural–Urban Inequities in Open Defecation across 20 Sub-Saharan African Countries (2022)

Equity profile	Classification criteria	Countries	Approx. population	Policy implication
High burden / extreme inequity	National prevalence ≥ 30 %; Absolute difference ≥ 40 pp	Eritrea, Chad, South Sudan, Niger, Burkina Faso, Central African Republic	≈ 86 M	Require large-scale rural infrastructure investment, governance reform, and nationwide sanitation campaigns.
Moderate burden / persistent inequity	National prevalence 10–29.9 %; Absolute difference ≥ 15 pp	Madagascar, Malawi, Mali, Mozambique, Liberia, Sierra Leone, Ethiopia, Togo, Guinea-Bissau, Democratic Republic of Congo	≈ 353 M	Need targeted subnational programmes combining behaviour-change communication and equity-focused resource allocation.
Low burden / residual inequity	National prevalence < 10 %; Absolute difference < 15 pp	Rwanda, Burundi, Gambia, Uganda	≈ 77 M	Maintain progress through sustained monitoring and inclusion of marginalised populations.

Together, the 20 countries analysed represent roughly 516 million people, of whom about 170 million still practise open defecation, largely concentrated in the high-burden group. The typology thus provides a practical framework for prioritising investments by equity profile rather than by average coverage alone.

Patterns indicate a west-central concentration of high-burden inequities (Chad, Niger, CAR, Eritrea, South Sudan) and relatively lower inequities in eastern and southern Africa (Rwanda, Uganda, Malawi). This distribution mirrors differences in governance capacity, decentralization, and community-led total-sanitation adoption, suggesting that institutional and social factors remain decisive determinants of sanitation equity across the region. A comparison with 2015 JMP data reveals that these rural-urban disparities are not only persistent but have widened in several key contexts. For instance, in Chad, the absolute rural-urban gap increased from 35.0% points in 2015 to 62.2 points in 2022. Conversely, in Sierra Leone, the gap narrowed from 25.2 to 21.7 points over the same period. This underscores that progress in reducing national prevalence does not automatically translate into narrower equity gaps and underlines the need for longitudinal equity tracking to ensure disparities are narrowing in line with SDG 6.2 targets.

The standardised metrics reveal that progress toward universal sanitation in Sub-Saharan Africa is constrained less by technology gaps than by distributional inequities. Eliminating rural disadvantage would reduce national open-defecation prevalence by as much as 40–60% in several countries, translating into tens of millions fewer people practising open defecation. These results highlight the need to embed equity metrics, particularly PAR and PAF into national monitoring systems so that efficiency and fairness advance together toward SDG 6.2.

## Discussion

Our cross-country analysis demonstrates that remaining sanitation deprivation in Sub-Saharan Africa is a problem of distribution as much as of coverage. While national averages are useful for monitoring progress, they conceal persistent and deep rural–urban gaps: rural populations account for the overwhelming share of continuing open defecation in the 20 countries analysed (Tables 1, 2 and 3). Applying standardized equity metrics (D, R, PAR, and PAF) shifts attention from who has access on average to who is being left behind and quantifies how much national progress depends on eliminating rural disadvantage. This reframing aligns with the WHO Health Inequality Monitoring Framework, which emphasizes that progress toward universal health and sanitation goals must be evaluated through the lens of distribution, not just averages [7]. In practical terms, eliminating rural disadvantage would yield substantial national gains. For instance, in Niger, eliminating the rural-urban disparity would reduce the national open-defecation prevalence from 65% to 9.6%, preventing roughly 11 million people from practising open defecation. This starkly illustrates the efficiency of equity-focused investments.

These findings confirm that achieving SDG 6.2 depends as much on equity-focused action as on technological or financial inputs. For instance, in Niger, eliminating the rural–urban disparity would reduce the national open-defecation prevalence from 65% to 9.6%, preventing roughly 11 million people from practising open defecation and starkly illustrating the efficiency of equity-focused investments.

Persistent rural-urban disparities reflect deep-rooted structural and institutional asymmetries that shape access to sanitation services across the region. Rural areas are typically marginalized in fiscal transfers, with local governments receiving unpredictable budgets or lacking dedicated funds for sanitation infrastructure and maintenance [13, 14]. Limited decentralization of water and sanitation governance further restricts the ability of rural authorities to plan and sustain interventions. In addition, supply-chain fragmentation driven by poor road access, higher transport costs, and weak private-sector participation raises the price of sanitation materials and services in rural settings, reinforcing inequities [13]. In many contexts, national programs have historically prioritized urban sewerage expansion over rural sanitation, entrenching investment bias [15].

Social and behavioural factors also play an important role. In several high-burden countries, entrenched norms surrounding defecation, perceptions of purity, and land ownership influence whether rural households demand or maintain latrines. Studies have shown that community-led total sanitation (CLTS) approaches, while successful in some regions, often fail to sustain gains when follow-up and financing mechanisms are weak [16, 17]. Environmental factors, including seasonal flooding or sandy soils, undermine latrine durability and discourage maintenance. Collectively, these constraints mean that rural sanitation challenges are systemic and multidimensional rooted not only in infrastructure gaps but also in the political economy of rural development [18].

### Programmatic implication

The typology developed in this study (Table 3) provides a policy-relevant framework for differentiating national strategies by equity profile rather than by average coverage alone. For high-burden and extreme-inequity countries such as Eritrea, Chad, South Sudan, Niger, Burkina Faso, and the Central African Republic, open defecation remains both widespread and structurally embedded. In these contexts, universal sanitation will require transformative investments that combine mass rural infrastructure programs, subsidized material supply chains, and national-scale social mobilization campaigns. Experience from Ethiopia’s One WASH programme and India’s Swachh Bharat Mission shows that sustained government leadership, coupled with community accountability, can accelerate equity gains when funding and local engagement are aligned [19].

For moderate-burden and persistent-inequity countries, including Madagascar, Malawi, Mali, Mozambique, Liberia, Sierra Leone, Ethiopia, Togo, Guinea-Bissau, and DRC, policies should focus on subnational targeting and differentiated financing. This group demonstrates significant rural-urban gaps despite moderate national prevalence. Equity-based budgeting, using disaggregated data to channel greater resources toward rural districts with large populations and slow progress, would yield the highest marginal gains [20]. Integration of sanitation with water, hygiene, and primary health services at the district level can also reduce inefficiencies and promote long-term behaviour change [21]. Partnerships between government, civil society, and community cooperatives have proven effective in bridging last-mile delivery barriers, particularly where government capacity is limited [22].

For low-burden and residual-inequity countries such as Rwanda, Burundi, Malawi, and Uganda, national coverage has improved dramatically, yet small inequities persist among the most remote or marginalized communities. These contexts should focus on maintaining gains through regular monitoring, quality assurance, and targeted inclusion of hard-to-reach populations. Sustaining rural progress requires continued investment in behaviour reinforcement and safe faecal sludge management to prevent reversion to open defecation. Importantly, even in these low-burden settings, the principle of “leaving no one behind” remains critical to meeting the SDG 6.2 equity mandate [23].

Traditional sanitation monitoring frameworks rely heavily on national averages that mask disparities. Our findings underscore the need to institutionalize standardized inequality metrics such as absolute difference, ratio, population attributable risk, and population attributable fraction within national and regional monitoring systems. Each metric serves a complementary role. D and R reveal the magnitude of the rural-urban gap, while PAR and PAF quantify the proportion of the national burden that could be averted if all groups achieved the same level as the best-performing subgroup. This approach transforms abstract equity goals into measurable, actionable indicators that can guide resource allocation.

Embedding these measures into national WASH monitoring and results frameworks would enhance accountability and inform performance-based financing. For example, a ministry could use PAF to estimate the national benefit of reducing rural inequity, then link budget allocations or donor disbursements to progress in narrowing the gap. PAR and PAF metrics also allow donors to compare equity efficiency across countries, rewarding those achieving both scale and fairness. The adoption of such metrics aligns with global calls for equity-sensitive monitoring under the WHO-UNICEF Joint Monitoring Programme and the WHO Health Inequality Data Repository.

Although our analysis focuses on rural-urban residence, the human consequences of open defecation are deeply gendered and intersectional. Women and girls in rural communities face heightened vulnerability to harassment, assault, and psychosocial stress due to lack of privacy. Open defecation also intersects with menstrual hygiene management, limiting participation in education and public life [24]. Moreover, women often bear the physical burden of constructing and maintaining latrines, despite limited decision-making power over household spending [25]. Addressing these inequities requires gender-responsive policies that ensure safe, well-lit, and private sanitation facilities, integrate menstrual hygiene management, and promote women’s leadership in community sanitation committees. Future monitoring should also disaggregate data by sex, disability, and wealth to capture intersectional disadvantage and ensure no subgroup is overlooked [26].

Geographic clustering of inequities, particularly across the Sahelian and Central African belt, suggests that governance capacity, decentralization effectiveness, and exposure to protracted crises strongly influence sanitation outcomes. In fragile contexts such as South Sudan or the Central African Republic, conflict and displacement erode infrastructure and disrupt local service delivery systems, amplifying rural disadvantage. Conversely, countries in Eastern and Southern Africa with stronger decentralization frameworks, such as Rwanda and Malawi, show evidence that sustained community mobilization and local accountability mechanisms can maintain low open-defecation rates [27]. These regional patterns highlight the importance of aligning sanitation policy with broader governance reforms. Strengthening local government financing systems, establishing rural sanitation funds, and integrating WASH into national poverty reduction strategies could accelerate convergence between rural and urban populations.

### Strengths and limitations

This study provides the most comprehensive cross-national assessment to date of rural-urban inequities in open defecation in Sub-Saharan Africa using harmonized 2022 JMP estimates. By applying standardized inequality metrics across 20 countries, it offers a rigorous and comparable measure of the magnitude and impact of inequity. The use of population-attributable risk and fraction measures allows quantification of the share of national burden that could be eliminated through equity-focused action, a perspective rarely applied in sanitation research.

Nonetheless, several limitations merit attention. First, the analysis is cross-sectional and cannot infer causality or temporal change. Longitudinal studies using multiple survey rounds would be needed to track progress over time. Second, the study relied on residence-based disaggregation; other equity dimensions such as wealth, gender, and disability were not analyzed, though they likely interact with rural disadvantage. Additionally, uncertainty intervals were not reported because several countries’ HEAT exports produced placeholder rather than valid estimates. Excluding these intervals does not affect interpretation, as the study aims to describe rather than test statistical differences. As explained in the Methods, these cases were removed during data cleaning to avoid misleading inference. As a result, findings should be interpreted as descriptive point estimates rather than statistically tested effects. Finally, while the equity typology is evidence-informed, it is rule-based and intended as a decision-support tool rather than a prescriptive classification. Furthermore, the cross-sectional nature of our analysis provides a crucial snapshot of inequities in 2022 but cannot assess whether these disparities are widening or narrowing. While broader regional reports indicate a general decline in open defecation alongside persistent inequities, future longitudinal research tracking these metrics over time is essential to evaluate the impact of equity-focused policies. National programs should adapt it to their own institutional and epidemiological contexts.

## Conclusions and future directions

Accelerating progress toward SDG 6.2 requires shifting from uniform, infrastructure-centered programming to equity-focused strategies that address systemic exclusion of rural populations. The findings demonstrate that eliminating rural disadvantage could substantially reduce national open-defecation prevalence and deliver both fairness and efficiency gains. Policymakers should therefore integrate standardized inequality metrics, particularly D, PAR, and PAF, into national monitoring frameworks and budget planning processes. Doing so would allow for transparent assessment of who benefits from progress and where investments yield the largest equity-adjusted returns.

Future research should expand the multidimensional scope of equity analysis by incorporating wealth, education, gender, and disability as cross-cutting stratifiers and by linking inequality measures to health outcomes such as diarrhoeal disease or child growth failure. Evaluations of equity-informed interventions such as targeted subsidies, conditional cash transfers, and decentralized financing models are also needed to determine which mechanisms most effectively reduce rural sanitation deficits. At the policy level, development partners and governments must commit to measuring progress not only by the number of facilities built but by the narrowing of inequity gaps.

Through operationalizing equity metrics within national and subnational planning, governments can transform the pursuit of universal sanitation into a catalyst for distributive justice. Ensuring that rural communities achieve parity in sanitation access is not merely a moral imperative, it is a prerequisite for sustainable development, gender equality, and public health resilience in Sub-Saharan Africa.

## Data Availability

The data underlying this study are publicly available from the WHO/UNICEF Joint Monitoring Programme through the WHO Health Inequality Data Repository. All data can be accessed at: https://whoequity-heat-1.share.connect.posit.cloud/#.

## Abbreviations

CI: Confidence Interval
DHS: Demographic and Health Survey
D: Difference (absolute inequality measure)
GDP: Gross Domestic Product
HEAT: Health Equity Assessment Toolkit
JMP: WHO/UNICEF Joint Monitoring Programme
N/A: Not Applicable
PAR: Population Attributable Risk
PAF: Population Attributable Fraction
R: Ratio (relative inequality measure)
SDG: Sustainable Development Goal
UNICEF: United Nations Children’s Fund
WHO: World Health Organization
